# Construction and prototype effect evaluation of a multi-agent collaborative system for operating room nursing

**DOI:** 10.3389/fdgth.2026.1770454

**Published:** 2026-06-03

**Authors:** Yifang Li, Jingfei Zou, Ling Wang, Rong Zhao, Jing Yuan

**Affiliations:** 1 Department of Operating Room, The Second Hospital of Nanjing, Nanjing, Jiangsu, China; 2 Department of Information Management, The Third People's Hospital of Chengdu, Chengdu, Sichuan, China; 3 Department of General Surgery, The Third People's Hospital of Chengdu, Sichuan, China

**Keywords:** automated machine learning, automatic knowledge construction, auxiliary decision-making, human-machine collaboration, multi-agent system, operating room management

## Abstract

**Objective:**

To develop an operating room intelligent collaborative management system, define its intelligent auxiliary role for nursing teams, and evaluate its efficacy in process optimization, efficiency improvement, and clinical acceptance.

**Methods:**

Guided by standards like the Guidelines for Operating Room Nursing Practice, five position-mapped agents (scheduling, resource, early warning, quality control, interaction) were designed. The system integrates a Graph RAG-based knowledge engine, MindsDB-powered AutoML prediction engine, and an innovative function to automatically construct/visualize knowledge graphs from uploaded nursing documents, with a user-friendly human-machine interface adapted to operating room settings. Simulated scenario tests and a 5-point Likert scale survey (86 medical staff) were conducted.

**Results:**

The system achieved success rates of 95.0% (resource conflicts), 90.0% (emergency insertion), 85.0% (equipment failures), and 90.0% (special coordination), with average solution generation time of 22.3–41.6 s. Overall nursing satisfaction was (4.32 ± 0.51) points, with top scores in “process optimization perception” (4.40 ± 0.48) and “decision support value” (4.35 ± 0.52).

**Conclusion:**

Integrating knowledge-driven and data-driven intelligence, the system enables automatic knowledge graph construction and updates. As an effective digital assistant for nursing collaboration, its nurse-centric, operating room-adapted design has gained wide clinical recognition, offering a human-machine collaboration solution for intelligent nursing management.

## 1 Introduction

Nursing stands as the backbone of healthcare delivery, yet it grapples with enduring challenges: overwhelming documentation burdens, cognitive overload, and the constant struggle to balance routine administrative tasks with high-quality, patient-centered care (1). For decades, health information technologies have attempted to mitigate these pain points, but many have inadvertently increased nurses’ screen time and administrative complexity, further straining an already pressured workforce (2). Artificial intelligence (AI), armed with advanced data analytics, automation, and real-time reasoning capabilities, has emerged as a promising solution—but its narrative in nursing has long been narrowly confined to documentation reduction (3).

Recent discourse among nursing informatics leaders, including chief nursing informatics officers (CNIOs) and clinical informatics experts, reveals that AI's potential extends far beyond easing administrative loads. As frontline advocates for both nurses and patients, these leaders are uniquely positioned to articulate AI's transformative role in reshaping nursing practices (4). Their insights highlight a critical paradigm shift: moving from using AI to “accelerate old workflows” to reimagining care delivery around activities that genuinely add value to both patients and nurses (5).

This study synthesizes expert perspectives from leading healthcare institutions to comprehensively explore AI's multifaceted impact on nursing. By analyzing key applications, core implementation principles, and future visions shared by these leaders, we aim to establish a holistic framework for understanding AI's potential (6). This framework centers human expertise, evidence-based practice, and value-driven care, emphasizing that AI should augment clinical decision-making, enhance patient safety, and empower nurses to focus on high-value care—rather than merely streamlining administrative tasks. Ultimately, this research seeks to guide the responsible integration of AI in nursing, fostering a future where technology serves as a trusted partner in advancing care quality and nursing well-being.

## 2 Methods

### 2.1 System construction basis and design: intelligent members of the nursing team

Guided by the “human-machine teamwork model,” the research team conducted in-depth investigations focusing on situation sharing, trust establishment, and capability complementarity among team members (5, 7). By systematically sorting out the workflows and collaboration needs of the nursing team, and referring to the Guidelines for Operating Room Nursing Practice, the consistency between the system logic and standard clinical processes was ensured (8). Adhering to the principle of “role correspondence and capability complementarity,” five core nursing positions were mapped to specialized agents (Table 1). The agents were designed as “digital partners” for nursing staff, with final judgment and execution rights retained by nurses to maintain their status as core decision-makers.

**Table 1:** Mapping between core nursing positions and collaborative agents in operating rooms.

Nursing Position/Team Role	Core Responsibilities and Collaboration Needs	Mapped Agent	Team Collaboration Positioning of the Agent
Head Nurse/Scheduler	Resource coordination, schedule formulation, emergency command	Surgical Scheduling Agent	Acts as a scheduling assistant to perform multi-objective dynamic scheduling and conflict simulation, providing optimized schemes for reference
Instrument/Circulating Nurse	Instrument counting, consumable preparation, inventory management	Resource Management Agent	Serves as a material steward for demand prediction and intelligent verification, recommending alternatives for shortages
Circulating/Anesthesia Nurse	Vital sign and equipment monitoring, abnormality detection	Risk Early Warning Agent	Functions as a monitoring specialist for multi-source data fusion and trend analysis, providing early warnings and potential causes
Quality Control/Infection Control Nurse	Operational compliance supervision, quality data analysis	Quality Control Agent	Acts as a compliance sentinel for automated screening and data extraction, marking potential deviations
Information Hub (Coordinator)	Cross-system information interaction and synchronization	Data Interaction Agent (Coordinator)	Serves as a communication hub to integrate information flow, interpret natural language instructions, and distribute tasks

### 2.2 Dual-Engine intelligent integration and automatic knowledge graph construction

To achieve reliable intelligent assistance, the system's core layer adopted a dual-engine architecture of “knowledge-driven” and “data-driven,” combined with an “large language models (LLMs)-assisted knowledge extraction and construction mechanism” to support the agent team's capabilities (8).

#### 2.2.1 Knowledge-driven engine (graph RAG) and LLM-assisted semantic

**Figure 1:**
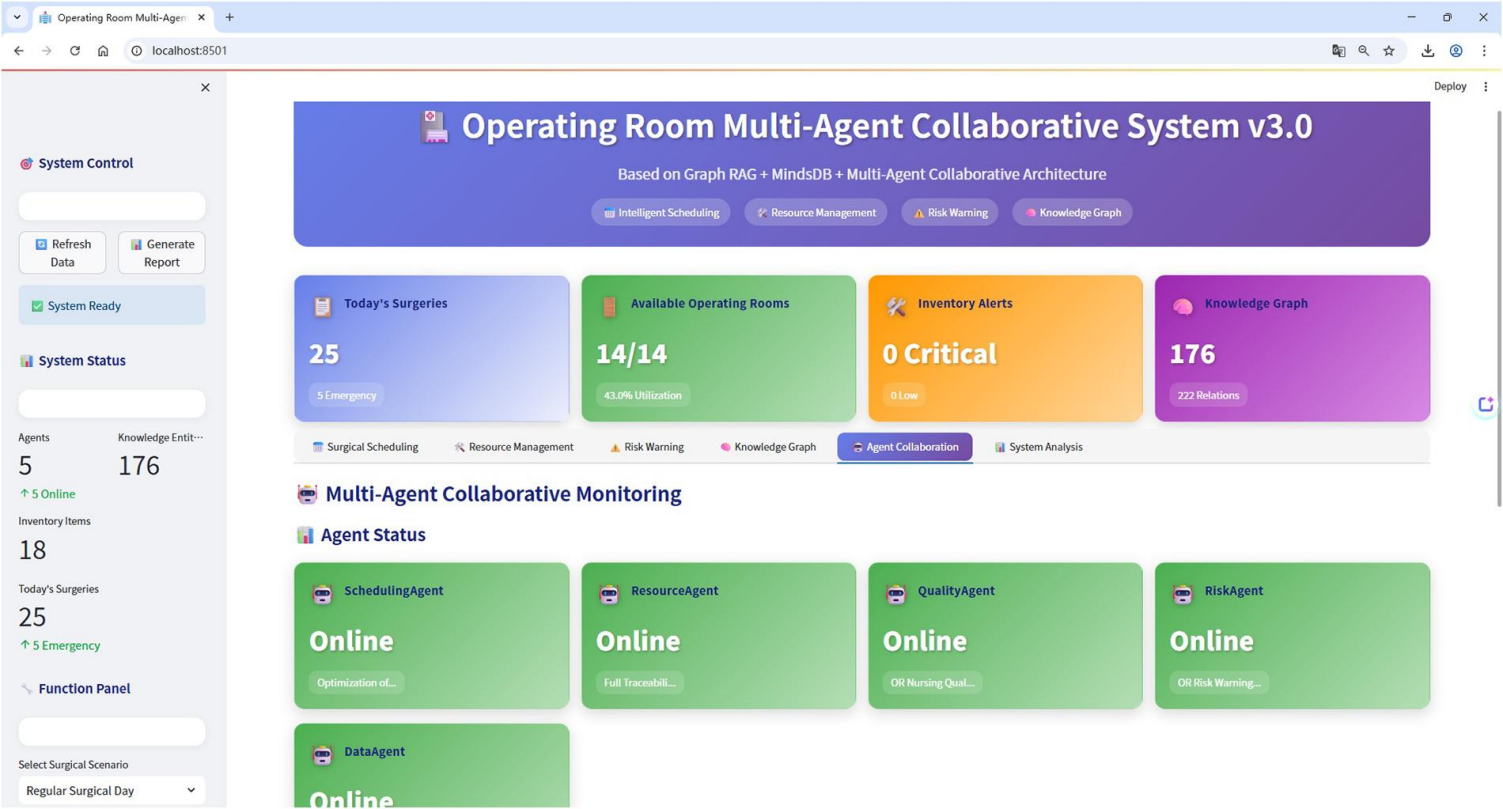
Intelligent scheduling and panoramic monitoring interface. Includes core modules such as daily surgical statistics, schedules, and duration predictions, supporting real-time monitoring of operating room (OR) status.

The Graph RAG engine provided interpretable and traceable decision-making basis, enabling automatic knowledge graph construction by uploading nursing documents. The system invoked large language models (LLMs) (8, 9) for in-depth semantic analysis and standardization, identifying core concepts such as nursing assessment and diagnosis, extracting implicit relationships and logic from texts, and integrating them into a structured nursing knowledge graph via a graph database (Figure 1). As shown in Figure 1, the system interface consists of several functional modules: (1) a surgical scheduling panel displaying daily surgeries, duration predictions, and real-time OR occupancy; (2) a resource status view showing availability of key equipment, consumables, and personnel; (3) an agent activity log that records requests, acknowledgments, and task delegations between agents and nurses in a timeline format; and (4) a decision support window where agents present analysis results, resource allocation plans, or conflict resolution options. This design ensures traceability of collaborative decision-making and aligns with the fast-paced, low-light environment of the operating room. This module manages and displays the structured communication flows between agents and, crucially, between the agents and the human nurse. It shows requests, acknowledgments, queries, and task delegations in a threaded or timeline format, ensuring traceability of the collaborative decision-making process. When agents generate analysis or suggestions (e.g., resource allocation plans, conflict resolution options, infection control reminders), they are presented here.

#### 2.2.2 Data-driven engine (MindsDB AutoML)

**Figure 2:**
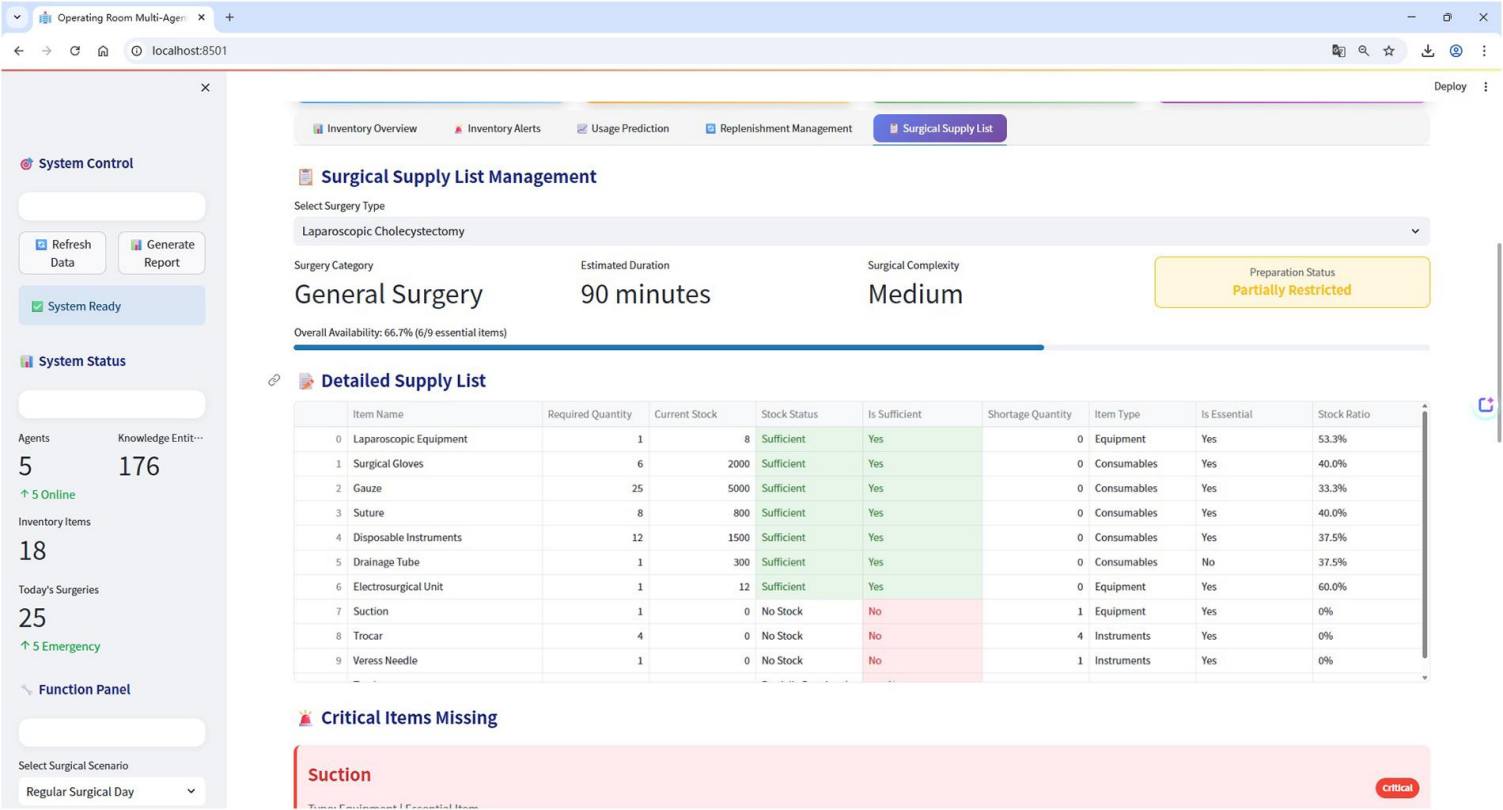
Operating room knowledge graph. Supports associated queries of surgical entities, risk tracing, and alternative recommendations, containing 176 knowledge entities and 222 relationships.

This engine excelled at rapidly and adaptively mining implicit rules from historical data to achieve accurate predictions, enhancing the team's ability to respond to dynamic changes (10). The MindsDB automated machine learning platform was seamlessly connected to the operating room data layer, automatically completing the entire workflow from data preprocessing and feature engineering to model selection, training, and deployment. For example, a time-series prediction model was used for surgical duration forecasting, and an XGBoost-based regression model for consumable demand prediction. Its AutoML feature significantly reduced technical barriers and maintenance costs, allowing the system to continuously optimize prediction accuracy with data accumulation (Figure 2).

Beyond the real-time interface, the system is designed to generate structured management-oriented reports for nursing directors, operating room managers, and hospital administrators to support operational decision-making.

##### 2.2.2.1 System decision support reports

The system incorporates a built-in reporting engine that generates five types of structured reports to support evidence-based decision-making by nursing managers:

Surgical scheduling efficiency report: outputs operating room occupancy rate, assesses the impact of emergency surgeries on elective procedures, and provides multi-objective optimization scheduling recommendations. Material consumption and inventory prediction report: based on an AutoML model, outputs demand forecasts for key consumables over the next 7–14 days and restocking recommendations. Surgical risk and quality monitoring report: provides complication risk stratification statistics, early warning event analyses, and key quality indicators such as infection prevention compliance rate. Multi-agent collaboration efficiency report: provides statistics on task processing volume, success rate, and response time for each agent. Surgeon preference analysis report: outputs preference coverage, material preparation consistency, and preference change history.

##### 2.2.2.2 Support for three Key decision factors

Nurse-patient ratio: real-time tracking of operating room count, nurse schedules, and patient acuity, automatically alerting imbalances and suggesting staffing adjustments. Surgery deferral: identifies resource shortages, equipment failures, or staff absences, and recommends surgical order adjustments or cross-department coordination.

##### 2.2.2.3 Interpretability for managers

Instead of directly displaying the complex knowledge graph (222 connections), the system provides: Visual dashboards (KPI cards, trend lines, red/yellow/green indicators); Intelligent summaries generated by large language models.

### 2.3 Human-machine interaction design centered on “auxiliary decision-making”

**Figure 3:**
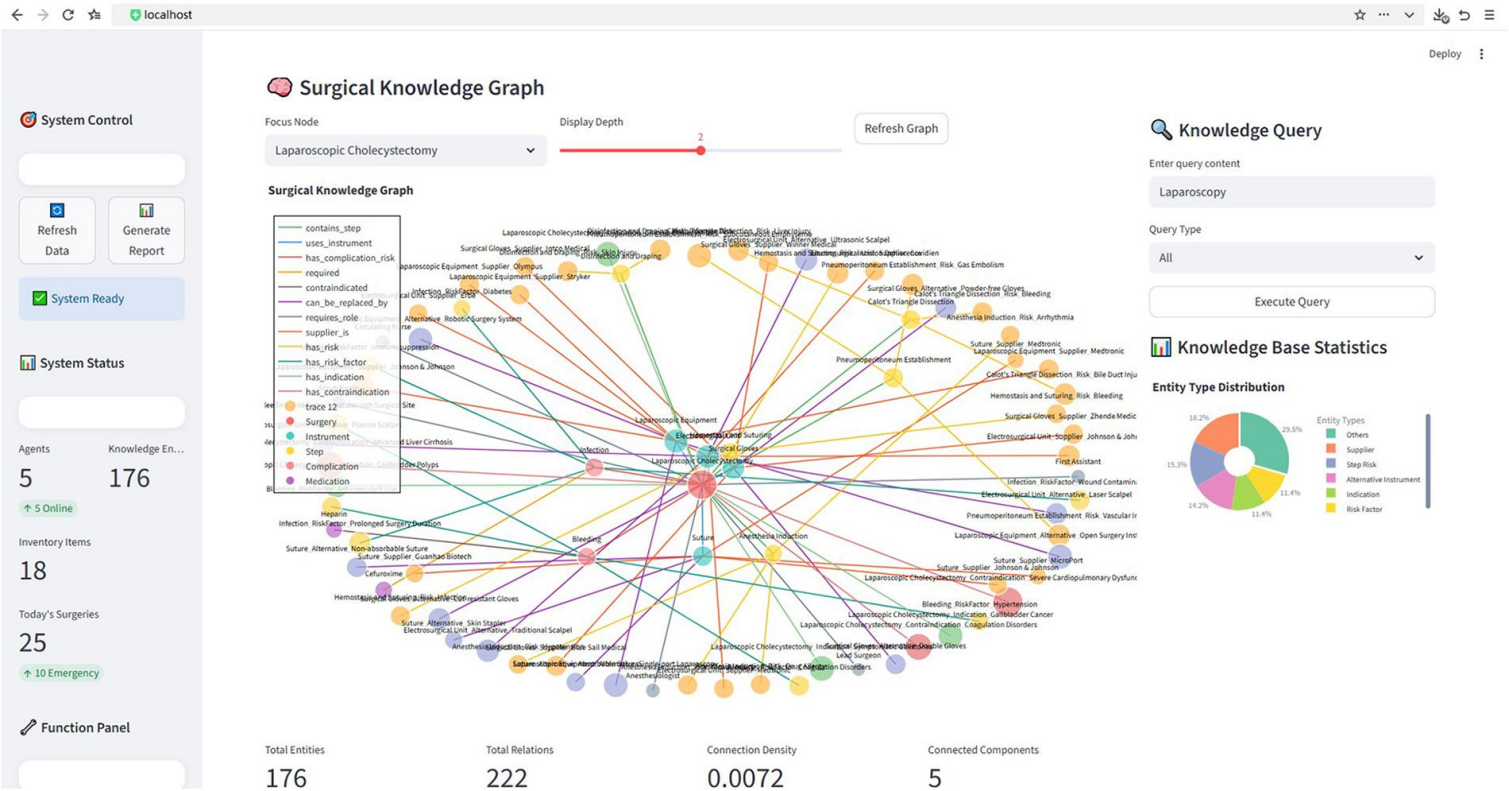
Prediction of operating room instrument usage. Predicts 7-day demand for 18 core items with an average confidence of 80%, supporting inventory early warnings and replenishment suggestions.

The application layer was designed to “promote efficient and tacit team cooperation,” with all interactions aligned to the goals of “clear presentation, simplified operation, and empowering people.” The interface adopted card-style layouts and highly recognizable colors to display surgical status, featured high-tolerance interactive elements for quick operations, and offered a dark mode to adapt to the operating room's low-light environment (Figure 3).

### 2.4 System verification and effect evaluation methods

#### 2.4.1 Operational testing based on simulated scenarios

Four typical complex scenarios were constructed using operating room nursing guidelines and expert experience. Each scenario was tested 20 times (*n* = 20), with evaluation indicators including: (1) problem-solving success rate (proportion of valid executable solutions); (2) average solution generation time (from scenario trigger to complete output); (3) blind evaluation scores (5-point scale) by 3 senior head nurses (≥10 years of experience) on clinical rationality and auxiliary value. The sample sizes used in the simulated scenario tests (*n* = 20 per scenario) and the user survey (*n* = 86), framed within the context of a feasibility and prototype evaluation study.

#### 2.4.2 User acceptance and usability survey

A self-designed 5-point Likert scale questionnaire (Cronbach's *α* = 0.89) (11) was distributed to 86 operating room nurses after prototype experience. The questionnaire covered core dimensions such as “decision support value,” “sense of system control,” and “sense of team collaboration” to assess nurses’ perceptions of the system as a “collaborative member.”

### 2.5 Statistical analysis

Quantitative data were analyzed using SPSS 26.0. Continuous variables (satisfaction scores, solution generation time) were presented as mean ± standard deviation (Mean ± SD). Differences in satisfaction across dimensions were analyzed using one-way ANOVA. The data's normality and homogeneity of variance were tested (using Shapiro–Wilk and Levene's tests) prior to conducting the one-way ANOVA for comparing satisfaction scores across dimensions, ensuring the test's assumptions were met.

## 3 Results

### 3.1 Simulated operation test results

To comprehensively verify the system's auxiliary performance in complex clinical scenarios, four typical high-frequency challenging scenarios were constructed based on the Guidelines for Operating Room Nursing Practice and expert consensus (resource conflict, emergency insertion, equipment failure, special coordination). Each scenario was tested 20 times (*n* = 20) with standardized simulated datasets, ensuring the consistency and validity of test conditions. The evaluation focused on three core indicators: problem-solving success rate, average scheme generation time, and expert blind evaluation score, to systematically assess the system's efficiency, rationality, and clinical applicability.

As shown in Table 2, the system demonstrated stable and reliable auxiliary capabilities across all test scenarios. The resource conflict scenario (multiple surgeries competing for key equipment/consumables) achieved the highest problem-solving success rate (95.0%), with 19 out of 20 tests generating valid executable solutions. This was attributed to the system's multi-objective dynamic scheduling algorithm, which comprehensively prioritizes surgical urgency, equipment compatibility, and alternative resource availability—for example, automatically recommending equipment replacement or schedule adjustment based on surgical type and patient condition. The average scheme generation time for this scenario was 28.7 s, reflecting the system's rapid response to resource allocation dilemmas.

**Table 2:** Simulation test results of system auxiliary performance (*n* = 20 runs/scenario).

Test Scenario	Number of Successful Auxiliary Schemes	Success Rate (%)	Average Scheme Generation Time (seconds)	Expert Score for Auxiliary Value (Mean ± SD)
A. Resource Conflict	19	95.0	28.7	4.4 ± 0.3
B. Emergency Insertion	18	90.0	22.3	4.2 ± 0.4
C. Equipment Failure	17	85.0	35.1	4.1 ± 0.3
D. Special Coordination	18	90.0	41.6	4.3 ± 0.2

The emergency insertion scenario (unplanned high-priority emergency surgeries requiring schedule adjustments) achieved a success rate of 90.0% (18/20 valid solutions) and the shortest average generation time (22.3 s). The system's advantage lies in its real-time grasp of operating room status (surgical progress, staff availability, equipment idle time) and rapid calculation of the optimal insertion window—for instance, identifying surgeries with short remaining duration or flexible schedules to minimize disruption to elective procedures. Expert evaluations noted that the system's recommendations balanced emergency response and elective surgery continuity, aligning with clinical emergency management principles.

The equipment failure scenario (sudden malfunction of critical devices such as anesthesia machines or electrocautery units during surgery) had the lowest success rate (85.0%, 17/20 valid solutions) and the longest average generation time (35.1 s). This was due to the complexity of equipment failure handling, which requires integrating equipment maintenance data, spare device availability, and surgical procedure adjustments. For example, if a backup device is unavailable, the system must recommend alternative surgical techniques or coordinate urgent equipment transfers, extending the decision-making cycle. However, expert scores remained above 4.1 points, indicating that even in complex failure scenarios, the system's solutions were clinically rational and actionable.

The special coordination scenario (process alignment issues in multi-disciplinary collaborative surgeries) achieved a success rate of 90.0% (18/20 valid solutions) with an average generation time of 41.6 s—the longest among all scenarios. This was because multi-disciplinary surgeries involve coordinated scheduling of multiple medical teams, specialized equipment, and sequential procedure alignment. The system's data interaction agent effectively integrated information from different departments, standardized procedure nodes, and identified potential bottlenecks (e.g., delayed arrival of specialized staff or mismatched equipment preparation). Expert evaluations highlighted the system's ability to streamline cross-departmental communication and reduce coordination errors, demonstrating high practical value for complex collaborative workflows.

Expert blind evaluation scores for all scenarios exceeded 4.1 points (5-point scale), with the resource conflict scenario scoring the highest (4.4 points). The evaluation panel—composed of 3 senior head nurses with over 10 years of operating room management experience—assessed solutions based on clinical compliance, risk control effectiveness, and operational feasibility. Feedback indicated that the system's recommendations strictly adhered to operating room nursing standards, adequately considered potential safety risks, and were easy to implement in clinical practice, confirming the system's strong clinical value.

### 3.2 User satisfaction and usability survey results

A self-designed 5-point Likert scale questionnaire was used to investigate the satisfaction and usability of the system among 86 operating room nurses (including circulating nurses, instrument nurses, and anesthesia nurses) from three tertiary hospitals. The questionnaire was pre-tested for reliability and validity (Cronbach's *α* = 0.89), covering five core dimensions: perception of process optimization, value of decision support, confidence in safety improvement, system interface usability, and clarity of information presentation. A total of 86 valid questionnaires were retrieved, with an effective recovery rate of 100%, ensuring the representativeness of survey results.

As presented in Table 3, the overall average satisfaction score of nursing staff was (4.32 ± 0.51) points, indicating a high level of acceptance and recognition of the system. The perception of process optimization dimension scored the highest (4.40 ± 0.48), reflecting the system's significant role in streamlining workflows—for example, automating equipment inventory checks, surgical schedule reminders, and compliance documentation, reducing nurses’ administrative burden and repetitive tasks. Many nurses reported that the system simplified complex coordination processes, allowing them to focus more on direct patient care.

**Table 3:** Scores of nurses’ satisfaction and usability evaluation on each dimension (*n* = 86).

Evaluation Dimension	Mean ± SD
Perception of Process Optimization	4.40 ± 0.48
Value of Decision Support	4.35 ± 0.52
Confidence in Safety Improvement	4.38 ± 0.50
System Interface Usability	4.30 ± 0.53
Clarity of Information Presentation	4.28 ± 0.55

The value of decision support dimension ranked second (4.35 ± 0.52), with nurses acknowledging the system's ability to provide multi-dimensional information and scientific recommendations. For instance, in risk monitoring, the system integrates patient vital signs, equipment status, and nursing guidelines to generate early warnings and potential intervention strategies, enhancing nurses’ decision-making confidence and accuracy. Open-ended feedback noted that the system's recommendations were “evidence-based and actionable,” complementing clinical experience without replacing professional judgment.

The confidence in safety improvement dimension scored (4.38 ± 0.50), highlighting the system's contribution to reducing clinical risks. Nurses reported that the system's real-time risk alerts (e.g., equipment failure warnings, surgical procedure deviations) and automated compliance checks helped identify potential safety hazards early, improving the overall safety of surgical care. The system interface usability (4.30 ± 0.53) and clarity of information presentation (4.28 ± 0.55) dimensions also received high scores, with nurses praising the interface's user-friendly design—card-style layouts, high-contrast colors, and quick operation buttons—which adapted to the fast-paced and low-light operating room environment.

## 4 Discussion

### 4.1 AI as a catalyst for nursing transformation

Our findings align with a growing body of literature recognizing AI's potential to transcend administrative efficiency and drive meaningful change in nursing (12, 13). By automating routine tasks, enhancing clinical decision support, and reducing cognitive load, AI empowers nurses to reclaim their core role as compassionate, expert caregivers,addressing longstanding challenges of burnout and task fragmentation (14).

The shift from “task-driven” to “knowledge-enabled” clinicians is particularly significant. As healthcare becomes more complex, AI serves as a “trusted assistant” (15), providing real-time insights and coaching that enhance nurse competence, especially for early-career staff (16). This not only improves care quality but also strengthens the nursing workforce by fostering professional growth and reducing turnover (6, 17, 18).

### 4.2 The imperative of centering nursing expertise

A key insight from leaders is the criticality of involving nurses in AI design and implementation. Too often, health technologies are developed without input from frontline staff, leading to tools that disrupt workflows and increase burden (2). By prioritizing evidence-based adoption and “humans-in-the-loop” oversight, AI can be tailored to nursing needs—ensuring it supports rather than undermines clinical practice (7).

This aligns with the principles of person-centered AI in healthcare, which emphasize that technology should adapt to human needs rather than the reverse (19). For nursing, this means AI tools must respect the irreplaceable value of human judgment, compassion, and contextual understanding—qualities that cannot be replicated by algorithms (7, 13, 20).

As a translational consideration for future development and potential deployment, securing formal intellectual property protection (e.g., through software copyright or patent registration, where applicable) for the system's unique architecture and algorithms is recommended, as publication itself does not confer such legal rights.

### 4.3 Addressing limitations and future directions

This study has limitations: insights are derived from a select group of China healthcare leaders, and perspectives may not reflect global or resource-constrained settings. Future research should include diverse nursing voices and quantitative evaluations of AI's impact on patient outcomes, nurse burnout, and care efficiency.

Additionally, challenges such as data privacy, algorithmic bias, and training needs must be addressed to ensure equitable AI adoption (20). Leaders should prioritize transparency in AI decision-making, provide ongoing education for nurses to build AI literacy, and establish governance frameworks that uphold ethical standards. Future work should also explore the integration of AI with other emerging technologies to create seamless, patient-centered care ecosystems. For example, combining predictive analytics with remote monitoring could enable proactive interventions for high-risk patients, reducing hospital readmissions and improving outcomes.

The high performance in structured, simulated scenarios is promising but requires validation in real-world, dynamic operating room environments. Future research should involve multi-center deployments, longitudinal studies to assess impact on hard clinical and operational outcomes, and testing under a wider variety of clinical situations.

## 5 Conclusions

Artificial intelligence in nursing is far more than a tool for reducing documentation—it is a catalyst for transforming care delivery, reimagining nursing roles, and enhancing patient safety. By focusing on four core domains and adhering to principles of evidence-based adoption, “humans-in-the-loop” oversight, and workflow reimagination, AI can empower nurses to deliver higher-quality, more compassionate care. The vision shared by nursing informatics leaders—of a care environment where intelligence serves as a trusted assistant—offers a roadmap for the future of nursing.

## Data Availability

The raw data supporting the conclusions of this article will be made available by the authors, without undue reservation.
