# Supplementary material for: Construction and prototype effect evaluation of a multi-agent collaborative system for operating room nursing
Source: Front Digit Health. 2026 Jun 3;8:1770454. doi: 10.3389/fdgth.2026.1770454 (PMC13272047; doi:10.3389/fdgth.2026.1770454)
Supplement: Supplementary file 1 [file Datasheet1.docx]

**Appendix: User Experience & Satisfaction Questionnaire**

Operating Room Intelligent Collaborative Management System

User Experience & Satisfaction Questionnaire

**Dear Nursing Colleagues:**

Thank you for participating in this evaluation of the Operating Room Intelligent Collaborative Management System. This questionnaire aims to understand your perception and acceptance of the system as an intelligent “collaborative partner” in clinical work. Your valuable feedback will help us further optimize the system to better support operating room care.

This survey is anonymous. All data will be used only for research and analysis. Please answer honestly based on your actual experience. Thank you for your support and cooperation.

**I. Basic Information**

1. Department: ______________
2. Role:

( ) Circulating nurse

( ) Scrub nurse

( ) Anesthesia nurse

( ) Head nurse / Nursing manager

( ) Other: ______________

1. Years of experience in operating room nursing:

( ) Less than 5 years

( ) 5–10 years

( ) More than 10 years

**II. System Satisfaction Evaluation**

Please rate each item according to your real experience.

Mark “√” in the corresponding box.

**Scale:**

1 = Strongly Disagree

2 = Disagree

3 = Neutral

4 = Agree

5 = Strongly Agree

| **Dimension & Items** | **Strongly Disagree** | **Disagree** | **Neutral** | **Agree** | **Strongly Agree** |
| --- | --- | --- | --- | --- | --- |
| **(A) Perceived Process Optimization** | 1 | 2 | 3 | 4 | 5 |
| 1. The system simplifies complex coordination processes (e.g., equipment allocation, communication). | 1 | 2 | 3 | 4 | 5 |
| 2. Automated functions (inventory, reminders) reduce my repetitive work. | 1 | 2 | 3 | 4 | 5 |
| 3. The system allows me to focus more on direct patient care. | 1 | 2 | 3 | 4 | 5 |
| **(B) Decision Support Value** | 1 | 2 | 3 | 4 | 5 |
| 4. Multi-dimensional information helps me make more accurate judgments. | 1 | 2 | 3 | 4 | 5 |
| 5. System recommendations improve my decision-making confidence. | 1 | 2 | 3 | 4 | 5 |
| 6. System suggestions are evidence-based and complement my clinical experience. | 1 | 2 | 3 | 4 | 5 |
| **(C) Safety Improvement Confidence** | 1 | 2 | 3 | 4 | 5 |
| 7. Real-time alerts help identify potential safety risks early. | 1 | 2 | 3 | 4 | 5 |
| 8. Automated compliance checks improve overall surgical safety. | 1 | 2 | 3 | 4 | 5 |
| 9. The system effectively helps me reduce clinical risks. | 1 | 2 | 3 | 4 | 5 |
| **(D) System Interface Usability** | 1 | 2 | 3 | 4 | 5 |
| 10. Interface design (layout, shortcuts) is convenient to use. | 1 | 2 | 3 | 4 | 5 |
| 11. System response is fast enough for the OR environment. | 1 | 2 | 3 | 4 | 5 |
| 12. The system is easy to learn without complex training. | 1 | 2 | 3 | 4 | 5 |
| **(E) Clarity of Information Presentation** | 1 | 2 | 3 | 4 | 5 |
| 13. Font, icons and colors are clear under OR lighting. | 1 | 2 | 3 | 4 | 5 |
| 14. Key information layout is clear and easy to locate quickly. | 1 | 2 | 3 | 4 | 5 |
| 15. Information (charts, knowledge graphs) is intuitive and understandable. | 1 | 2 | 3 | 4 | 5 |

**III. Open Questions**

- In which aspects does the system help your work most? Please give examples.
- What suggestions do you have for future improvement of the system?
